# Mental health problems among individuals with persistent health challenges from adolescence to young adulthood: a population-based longitudinal study in Norway

**DOI:** 10.1186/s12889-016-3655-z

**Published:** 2016-09-15

**Authors:** Sølvi Helseth, Dawit Shawel Abebe, Randi Andenæs

**Affiliations:** 1 Department of Nursing and Health Promotion, Faculty of Health, Oslo and Akershus University College, Oslo, Norway; 2 Centre for Welfare and Labour Research, NOVA, Oslo and Akershus University College, Oslo, Norway

**Keywords:** Longitudinal survey, Persistent health challenges, Mental health, Adolescents, Young adults

## Abstract

**Background:**

Persistent health challenges are increasing throughout the world. It has been shown that adolescents with persistent health challenges are at greater risk of having mental health problems than their healthy peers. However, these studies are mainly cross-sectional, and little is known about the transition to adulthood. Thus, the aim of this study was to examine how mental health problems in adolescents and young adults with persistent health challenges vary during adolescence and in the transition to young adulthood.

**Methods:**

The study used longitudinal and time-series data from the “Young in Norway” study. A sample of adolescents was prospectively followed from adolescence to young adulthood with measures at four different time points (*n* = 3,087; T1–T4): 2921 adolescents (12–19 years) participated at T1 and T2, while 2448 young adults participated at T3 and T4. Persistent health challenges, age, gender, mental health problems and parental socio-economic status were measured in the longitudinal survey. Regression models were applied to estimate associations between persistent health challenges (understood as having a chronic health condition or disability) and mental health problems during adolescence and young adulthood. Different models were tested for chronic health conditions and disability.

**Results:**

Adolescents with disability had higher scores for depressive and anxiety symptoms, loneliness and self-concept instability, and lower scores for self-worth, appearance satisfaction, scholastic competence and social acceptance compared with adolescents without disability. In young adulthood, there were also significant associations between disability and most mental health problems. The longitudinal associations between chronic health conditions and mental health problems during adolescence and young adulthood showed that significant associations between chronic health conditions and mental health problems were only found during adolescence.

**Conclusions:**

This longitudinal survey revealed that on average, adolescents with disability had more mental health problems than those with a chronic health condition. In addition, the problems followed into adulthood for adolescents with disability. Thus, disability seems to be a much higher risk factor for developing and maintaining mental health problems than having a chronic health condition. These findings need to be followed up in further studies.

## Background

The incidence and prevalence of persistent health challenges are increasing throughout the world, and chronic conditions and disability are emerging as major health problems in society [1]. ‘Persistent health challenges’ is a collective term pointing to health conditions and disability that affect daily living in various ways and calls for attention from self, caregivers and the health-care system to reduce the negative impact of the condition and secure optimal health and functioning. The World Health Organization (WHO) has proposed a framework to understand and measure disability, which captures the level of functioning in six domains of life [2]: 1. Cognition (understanding and communicating); 2. Mobility (moving and getting around); 3. Self-care (attending to one’s hygiene, dressing, eating and staying alone); 4. Getting along (interacting with other people); 5. Life activities (domestic responsibilities, leisure, work and school); 6. Participation (joining in community activities, participating in society). Diseases, injuries or congenital conditions might affect all of these areas or just one or two. A person’s functional ability and level of disability will, depending on how the condition is perceived and coped with, presumably have an impact on the mental health and quality of life of that person.

The prevalence of persistent health challenges among adolescents is difficult to assess because of the lack of quality data specifically on this age group and the diversity in methodology and definitions used [1]. However, it is reasonable to estimate that 10–15 % of Norwegian adolescents have significant ongoing health-care needs related to chronic health conditions or disability [1, 3, 4]. Persistent health challenges impact adolescents’ lives in various ways depending on the nature and severity of the challenge. However, adolescents living with a persistent health challenge, understood as a chronic health condition or disability, have been shown to be subject to reduced health-related quality of life [5–9]. In addition, it has been shown that emotional, behavioural and developmental problems are associated with persistent health challenges during adolescence [10–12]. Persistent health challenges may hinder the development of independence, social functioning, peer relationships and self-esteem, all of which are particularly important during adolescence and the transition to young adulthood [13]. Living with a persistent health challenge can be strenuous in itself, can lead to absence from school and poor school performance, and is also associated with emotional behavioural problems such as anxiety and depression, which again can result in social withdrawal [1, 14–16]. School attendance is important for participation in the academic, social and cultural communities, which provide opportunities for the future. It is important to be aware that the negative effects of such developmental experiences can follow into adulthood and affect learning and work achievements through the transition from adolescence to young adulthood [17–19].

So far, research has shown that adolescents with persistent health challenges are at greater risk of having mental health problems than their healthy peers [10, 11, 15]. However, most studies on this issue have been cross-sectional, and there is little information regarding the development of mental health problems over time for adolescents and young adults with persistent health challenges. To better understand the challenges adolescents and young adults living with a chronic health condition or disability face, and how they vary over time, we analysed data from a longitudinal Norwegian study “Young in Norway”. Greater knowledge of these matters will enable increased awareness of the associations between mental health problems and persistent health challenges, specifically how they develop with increasing age, and further, may warrant interventions that target these problems.

In the present study, we targeted adolescents and young adults living with diabetes, asthma or allergy, and selected disabilities (i.e., learning problems and physical impairments). Diabetes and asthma/allergy are well-known chronic health conditions. The incidence of asthma is measured in different ways, and varies with how asthma is defined and which methods are used to measure it. Figures from Norway show that the incidence is roughly the same as in other Nordic countries, but lower than in English-speaking countries. According to Norwegian research, between 5 and 11 % of 10-year-olds have asthma, which is more frequent in early childhood than in adolescence and adulthood. About half of the children have “grown out of” the disease by the age of 10 [3]. In Norway, type 1 diabetes among children and adolescents is increasing, with the incidence currently at 28/100,000 per year in children below the age of 15 [4]. Because of the large variety in definitions of disability, we were not able to find numbers describing the prevalence of the selected forms of disability in the present study.

In this paper, we compared mental health status between adolescents with and without persistent health challenges (diabetes, asthma or allergy, and selected disabilities) during adolescence and young adulthood. However, the main aim of this study was to examine how mental health problems vary during adolescence and in the transition to young adulthood in individuals with persistent health challenges. In addition, it aimed to investigate whether gender and parental socio-economic status (SES) could contribute to explaining variations in mental health problems.

## Methods

### Procedure and participants

**Fig. 1:**
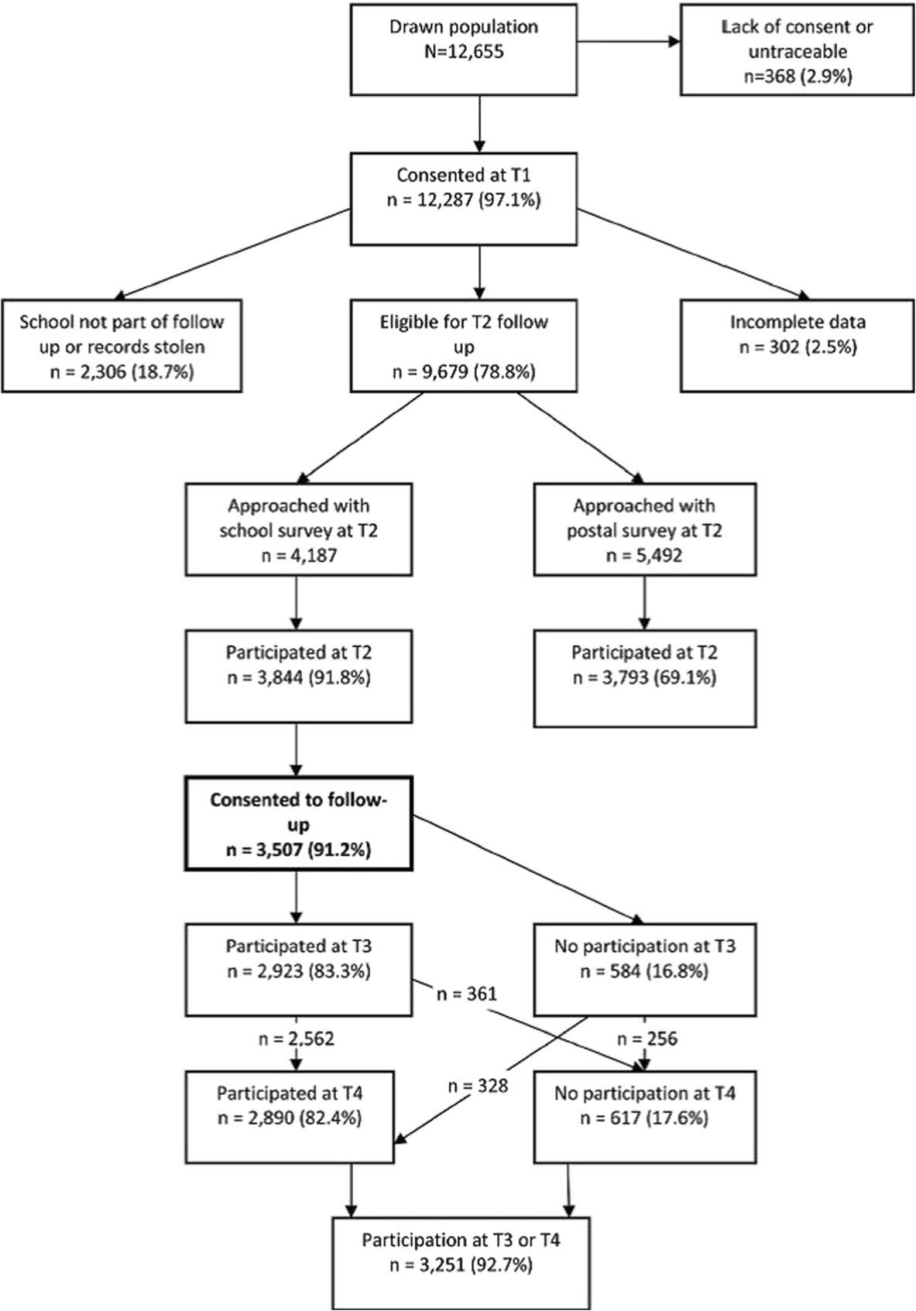
A flow chart of participation in the Young in Norway study

Data were analysed from the Norwegian longitudinal study “Young in Norway”, which was conducted at four time points: 1992 (T1), 1994 (T2), 1999 (T3) and 2005 (T4) (Fig. 1). The initial sample at T1 was composed of 12,655 students in grades 7–12 (12–20 years of age) at 67 representative schools in Norway, with each grade being equally represented. Every school in the country was included in the register from which the schools were selected, and the sample was stratified according to geographical region and school size, which in Norway is closely related to the degree of urbanization. Each school’s sampling probability was proportional to the number of students at the school, thus providing an equal probability of selection for each student. The response rate at T1 was 97 % (*n* = 12,287).

In 1994, three of the participating schools at T1 were not part of the follow-up study (T2; ages 14–22), and at another school, a burglary in the school’s archives resulted in the loss of the project’s identification records. In total, 9,679 students at 63 schools were eligible to complete the T2 questionnaire. Because a considerable proportion of the students had completed their three-year track at the junior or senior high school they were attending at T1, the subjects who were no longer at the same school at T2 received the questionnaire by mail. For this group, the response rate was 68 % (*n* = 3,783), whereas those still at their original schools had a response rate of 92 % (*n* = 4,187). The overall response rate at T2 was 79 %.

At T3, only students who had completed the questionnaire in school at T2 (*n* = 3,844) were followed up because of the comparatively lower response rate among those receiving the questionnaire by mail. As such, those who responded by mail at T2 (*n* = 3,783) were not included in the follow-ups at T3 and T4. Because the survey was originally planned as a two-wave study, informed consent had to be obtained again at T2 for follow-up at T3. Of the total number of consenting individuals at T2 (*n* = 3,507, 91.2 %), 2,923 (83.8 %) responded to the questionnaire that they received by mail at T3 (ages 19–28), representing an overall response rate of 68 %.

In 2005 (T4), all persons who had consented to the follow-up at T2 were again invited to participate (ages 25–34). In total, 2,890 of the 3,507 potential participants (82.4 %) completed the questionnaire, resulting in an overall response rate of 67 %. See Fig. 1 for the flow of participation in the Young in Norway study.

For the purposes of this study, to examine longitudinal associations between mental health status and persistent health challenges during adolescence and young adulthood, those who had responded at T1 and T2, and T3 or T4 were first selected (*n* = 3,087, 45.5 % males and 54.5 % females). The sample populations were further limited to represent the adolescence period of T1–T2 (*n* = 2,921; ages 12–19 years) and the young adulthood period of T3–T4 (*n* = 2,448; ages 20–34 years).

In addition, even though a large proportion of the sample that did not respond to the questionnaires at T3 and T4 were planned non-responders, analyses were conducted to explore the potential impact of variables on attrition. More specifically, we performed a multiple logistic regression to investigate whether variables at T1 predicted drop-out at T2, T3 or T4. The results of these analyses revealed that older age, male gender, more occasions of alcohol intoxication over the past year and higher perceived parental overprotectiveness significantly predicted higher chances of drop-out at T2, T3 or T4 (*p* < 0.05). Lower scores for parental care and loneliness also predicted drop-out (*p* < 0.05).

### Measures

All variables were based on self-report questionnaires. Persistent health challenges refer to chronic health conditions and disabilities. Participants at T1 and T2 were asked to indicate any disease or injury that had lasted for more than half a year and limited their daily activities. Having asthma at T1 or asthma/allergy at T2 or diabetes at T2 were included to indicate chronic health problems. Because the study questionnaire at T2 combined the presence of asthma or allergy into a single item, we could not separate those who reported asthma only. For disability, participants reported difficulties with speaking, reading and writing, and having physical disability at T1, and indicated being dyslectic, and having impaired vision, hearing and movement disability at T2. The presence of one of these problems at T1 or T2 was defined as disability.

### Mental health variables

Negative affectivity, including symptoms of depression and anxiety, was measured with a 12-item short version of the Hopkins Symptom Checklist [20]. Using a response scale of 1–4, participants were asked to restrict their ratings to the preceding week. Mean scores were calculated, with high scores indicating high levels of negative affectivity. The scale revealed satisfactory internal consistency on all occasions, with α values of 0.87, 0.88, 0.89 and 0.89 at T1, T2, T3 and T4, respectively.

General self-worth was measured using the Global Self-Worth Subscale from a revised version of Harter’s Self-Perception Profile for Adolescents [21, 22]. Five items assess how an adolescent views him- or herself, with the response options ranging from 1 (“corresponds very poorly”) to 4 (“corresponds very well”). Higher mean scores reflect high self-worth. The scale had an acceptable internal consistency on all occasions: 0.70, 0.75, 0.75 and 0.78 at T1, T2, T3 and T4, respectively.

Appearance satisfaction was assessed by the Body Areas Satisfaction Scale (BASS) [23]. The scale consists of seven items rating the individual’s level of satisfaction with the following seven body areas: face, lower torso, mid-torso, upper torso, muscle tone, weight and height. Responses were rated on a five-point Likert scale and varied from 1 (“very dissatisfied”) to 5 (“very satisfied”). A mean score was computed, with high scores indicating a high level of satisfaction. The BASS scale showed acceptable validity and high test–retest reliability; one-month test–retest coefficients were 0.86 for males and 0.74 for females [24]. The scale demonstrated good internal consistency at each survey point with α = 0.80, 0.81, 0.82 and 0.82 at T1, T2, T3 and T4, respectively.

Loneliness was measured by a five-item version of the UCLA Loneliness Scale [25], with each item having response options ranging from 1 (“never”) to 4 (“often”). This shortened version of the scale has been used as an adequate alternative to the longer 20-item version of the loneliness scale [26]. A higher mean score reflects greater loneliness. The five-item scale exhibited somewhat low internal consistency at T1 (α = 0.65), but acceptable α-values at the remaining three time points, with α = 0.72, 0.76 and 0.78 at T2, T3 and T4, respectively.

Alcohol intoxication was measured by asking participants to indicate how often they had “drunk so much that you felt clearly intoxicated” during the preceding 12 months, and the response scale ranged from 1 (“never”) to 6 (“more than 50 times”). Such self-report measures of substance use behaviours have showed good validity and reliability [27, 28]. Test–retest correlations were high and ranged from 0.72 to 0.83 [27]. High mean scores indicate a high level of alcohol use.

Self-concept instability was measured using a revised version of Rosenberg’s Stability of Self Scale [29], which has four items, each with a response scale ranging from 1 to 4. High scores indicate an unstable perception of self [29, 30]. The internal consistency was satisfactory at each survey point: 0.81, 0.86, 0.88 and 0.89 at T1, T2, T3 and T4, respectively.

Scholastic competence and social acceptance were measured by the revised version of Self-Perception Profile for Adolescents [21, 22]. Each subscale included five items with response options ranging from 1 (“corresponds very poorly”) to 4 (“corresponds very well”). High mean scores indicate a high level of perceived self-concept towards scholastic competence and social acceptance. Because the scholastic competence subscale was not assessed at T3 or T4, we only used it during adolescence. The internal consistency of the scholastic competence subscale was 0.68 and 0.70 at T1 and T2, respectively, and for the social acceptance subscale it was 0.70, 0.74, 0.78 and 0.82 at T1, T2, T3 and T4, respectively.

Age and gender were recorded in all surveys. Male was coded as “0” and female as “1”. Parental SES was determined from the adolescents’ reports of maternal and paternal occupation at T1. Occupations were categorized according to the ISCO-88 classification [31]. All parents were assigned to one of five categories; 1 (manual workers), 2 (primary industry workers), 3 (lower professional workers), 4 (middle professional workers), and 5 (professional leaders). The combined scores of both parents’ categories were used to indicate parental SES, and also used as a dichotomy variable to indicate high parental SES (middle professional workers and professional leaders) and low parental SES (other categories).

### Statistical analysis

Linear random intercept models were employed to investigate associations between mental health problems and persistent health challenges during adolescence and young adulthood. Such models have the great advantage of controlling for dependence among repeated responses of a subject [32]. The random intercept explains the average variability between subjects at starting age, and the random slope refers to the average variability between subjects over age. The fixed part of the model, explaining changes in the mean scores of mental health problems over age groups, is presented and discussed in this paper.

We performed a series of regression models during adolescence and young adulthood, where each mental health problem was added as an outcome, and persistent health challenges added as a covariate, and controlled for gender, age and parental SES. Further, in order to investigate risk differences across gender and parental SES during adolescence and young adulthood, the two-way interaction terms persistent health challenges * gender/persistent health challenges * parental SES were separately added into multiple regression models. We then compared average risk differences (i.e., using margins and nlcom commands) for mental health problems between females and males, and high versus low parental SES. For all analyses, a *P* value < 0.05 was considered statistically significant. Maximum likelihood estimates were applied. We used Stata SE/11 for Windows for all analyses.

## Results

### Descriptive summary

In this study, 12.9 % (*n* = 400; 52 % females and 48 % males) and 11.6 % (*n* = 359; 60 % females and 40 % males) reported disability and chronic health conditions at T1 and T2, respectively. Table 1 provides supplementary information on the prevalence of different forms of disability included. This shows that among individuals with disability in this study, 50 % have one kind of learning disability and 50 % have a physical disability. The descriptive summary of study variables is presented in Table 2. Adolescents (T1 or T2) reported higher symptoms of depression and anxiety, loneliness and self-concept instability than young adults (T3 or T4), while young adults reported more alcohol intoxication and social acceptance than adolescents. Analyses of variance with Bonferroni corrections were used for mean comparisons across time points (see Table 1).

**Table 1:** Prevalence of different forms of disabilities reported at T1 and T2

Forms of disabilities	Number	Percent
T1		
Difficulty with speaking	49	1.64
Difficulty with reading and writing	187	6.25
Having physical disability	53	1.77
T2		
Being dyslectic	46	1.47
Having impaired vision	143	4.58
Having impaired hearing	22	0.70
Having movement disability	46	1.47
Having one of learning disabilities at T1/T2	230	7.88
Having one of physical disabilities at T1/T2	232	7.75

*T* survey time point

**Table 2:** Descriptive summary of the study participants over time (*n* = 3,087)

Variables	Time 1	Time 2	Time 3	Time 4	Bonferroni test
	M (SD)	M (SD)	M (SD)	M (SD)	(*P* < 0.05)
Age	14.89 (1.7)	16.38 (1.70)	21.84 (1.76)	28.35 (1.70)	-
Depressive symptoms	1.74 (0.56)	1.76 (0.58)	1.72 (0.59)	1.59 (0.57)	c, e, f
Anxiety symptoms	1.47 (0.46)	1.47 (0.47)	1.42 (0.46)	1.35 (0.43)	b, c, d, e, f
Loneliness	1.87 (0.54)	1.83 (0.55)	1.81 (0.50)	1.77 (0.50)	b, c, e,
Self-worth	2.88 (0.55)	2.55 (0.33)	2.57 (0.31)	2.54 (0.29)	a, b, c, d, f
Appearance satisfaction	3.43 (0.65)	3.46 (0.66)	3.49 (0.65)	3.49 (0.62)	c
Scholastic competence	2.88 (0.51)	2.91 (0.54)	-	-	a
Social acceptance	3.07 (0.50)	3.16 (0.51)	3.21 (0.51)	3.21 (0.52)	a, b, c, d, e
Alcohol intoxication	1.88 (1.41)	2.48 (1.60)	3.76 (1.52)	3.47 (1.54)	a, b, c, d, e, f
Self-concept instability	2.62 (0.65)	2.49 (0.70)	2.33 (0.75)	2.13 (0.73)	a, b, c, d, e, f

*M* mean, *SD* standard deviation

a: significant mean difference between Time 1 and Time 2; b: significant mean difference between Time 1 and Time 3; c: significant mean difference between Time 1 and Time 4; d: significant mean difference between Time 2 and Time 3; e: significant mean difference between Time 2 and Time 4; f: significant mean difference between Time 3 and Time 4

### Longitudinal associations between disability and mental health problems

As shown in Table 3, in order to examine associations between disability and mental health problems, we conducted a series of linear random intercept models (fixed effects) during adolescence and young adulthood. The regression estimates in Table 3 were adjusted for gender, age and parental SES. Except for alcohol intoxication, significant associations were found between all mental health problems and disability during adolescence (*P* < 0.05); adolescents with disability had higher scores for depressive and anxiety symptoms, loneliness and self-concept instability, and lower scores for self-worth, appearance satisfaction, scholastic competence and social acceptance compared with adolescents without disability.

**Table 3:** Linear random intercept models for longitudinal associations between disability and mental health problems during adolescence and young adulthood

Mental health problems	Adolescence (*n* = 2,921; 12–19 yrs) β (se)	Young adulthood (*n* = 2,448; 20–34 yrs) β (se)
Depressive symptoms	0.13 (0.03)***	0.09 (0.03)*
Anxiety symptoms	0.10 (0.02)***	0.10 (0.02)***
Loneliness	0.16 (0.03)***	0.11 (0.03)***
Self-worth	−0.09 (0.02)***	−0.01 (0.01)
Appearance satisfaction	−0.13 (0.03)***	−0.11 (0.03)**
Scholastic competence^a^	−0.17 (0.02)***	
Social acceptance	−0.08 (0.02)***	−0.10 (0.03)**
Alcohol intoxication	−0.01 (0.06)	−0.18 (0.08)*
Self-concept instability	0.08 (0.03)**	0.07 (0.04)

*β* regression estimates adjusted for gender, age and parental, *SES* se: standard error

* *P* < 0.05; ** *P* < 0.01; *** *P* < 0.001

^a^Scholastic competence was only measured during adolescence

In young adulthood, there were also significant associations between disability and most mental health problems, except that associations with self-worth and self-concept instability were non-significant (*P* > 0.05), and young adults with disability reported significantly lower alcohol intoxication than those without disability.

We then investigated whether gender and parental SES moderate associations between disability and mental health problems. Interaction terms between gender and disability were significant for loneliness (β = −0.11, *P* < 0.05) and scholastic competence (β = 0.13, *P* < 0.01) during adolescence, indicating that adolescent females with disability had a lower score for loneliness as well as higher scholastic competence. As to interaction terms between parental SES and disability, significant associations were found for depressive symptoms (β = 0.11, *P* < 0.05) and self-worth (β = −0.09, *P* < 0.05) during adolescence, revealing that adolescents with disability and low parental SES had more depressive symptoms and lower self-worth. Non-significant interaction terms between disability and gender or parental SES were found during young adulthood.

Results presented in Table 3 are, with a few exceptions, consistent when analyses are stratified by learning and physical disabilities.

### Longitudinal associations between chronic health conditions and mental health problems

Table 4 shows the results of longitudinal associations between chronic health conditions and mental health problems during adolescence and young adulthood. A similar modelling approach and analysis to those used for the results shown in Table 2 were applied. Significant associations between chronic health conditions and mental health problems were found only during adolescence, where adolescents with chronic health conditions reported more symptoms of depression and anxiety, and lower appearance satisfaction than those without chronic health conditions. Associations between chronic health conditions and mental health problems during young adulthood were not significant (*P* > 0.05). Moreover, there were no significant interaction terms between chronic health conditions and gender or parental SES.

**Table 4:** Linear random intercept models for longitudinal associations between chronic health problems and mental health problems during adolescence and young adulthood

Mental health problems	Adolescence (*n* = 2,921; 12–19 yrs) β (se)	Young adulthood (*n* = 2,448; 20–34 yrs) β (se)
Depressive symptoms	0.10 (0.02)***	0.02 (0.02)
Anxiety symptoms	0.08 (0.02)***	0.03 (0.02)
Loneliness	0.02 (0.02)	0.02 (0.02)
Self-worth	−0.02 (0.02)	−0.02 (0.02)
Appearance satisfaction	−0.06 (0.02)*	−0.02 (0.03)
Scholastic competence^a^	−0.03 (0.02)	
Social acceptance	−0.03 (0.02)	−0.03 (0.02)
Alcohol intoxication	0.01 (0.06)	−0.04 (0.07)
Self-concept instability	0.01 (0.02)	0.01 (0.03)

*β* regression estimates adjusted for gender, age and parental, *SES* se: standard error

* *P* < 0.05; ** *P* < 0.01; *** *P* < 0.001

^a^Scholastic competence was only measured during adolescence

## Discussion

The findings of this study clearly reveal that adolescents living with persistent health challenges are more subject to mental health problems than their healthy peers. Adolescents with disability scored significantly worse than their peers without disability on the majority of the scales used to measure mental health problems. Adolescents with a chronic health condition also had significantly more mental health problems than their healthy peers, but only on a few scales; anxiety symptoms, depressive symptoms and appearance satisfaction. Different patterns of development over time and the presence of mental health problems were seen between young people with disability and young people with a chronic health condition.

The finding that adolescents with disabilities are at greater risk of having mental health problems than their non-disabled peers is consistent with the literature in the field [33–35]. For instance, a recent Swedish study showed that adolescents with impairments, particularly girls and those with multiple impairments, have considerably worse mental health than others [34].

Our study indicates that parental SES moderates associations between disability and mental health problems in adolescents with disability. So far, few studies exist about modifiable factors that can predict the development of mental health problems. However, a large European longitudinal study of adolescents with cerebral palsy showed that psychological problems in early childhood persisted into adolescence [36]. Further, early childhood factors, such as psychological problems and parental stress, predicted adolescent participation largely through their persistence into adolescence [36]. Parental stress was not measured in our study; however, significant associations were found for depressive symptoms and self-worth during adolescence, revealing that adolescents with disability and low parental SES had more depressive symptoms and lower self-worth. It could be assumed that low parental SES might be associated with parental stress, and thus might also be associated with social participation; however, this needs further study. Honey and colleagues [37] suggested, as a result of their study on the impact of social conditions on the mental health of young people with disability, that disability represents a potential adversity that might be improved by the effects of wealth and social support.

Many adolescents living with disability experience restricted participation in life situations ranging from leisure activities to education and social roles [38]. Transitioning into adulthood, they remain at higher risk of social disadvantage depending on the type and severity of the impairment [39]. In the present study, mental health problems in adolescence continued into early adulthood in all areas except for self-worth and stability of self. One explanation for this might be that adolescence is a challenging period for everybody, but in particular for young people with disability who are adapting to the changes of puberty and forming their identities [40]. On reaching adulthood, these individuals have, to a greater extent, come to terms with who they are. However, our findings show that young adults with disability continue to be lonelier, experience more anxiety and depression, are more dissatisfied with their appearance and experience lower social acceptance then those without disability. Mental health problems in individuals with disability are further associated with participation in life [36] in terms of independent living, establishing a family, educational attainment, employment and lower annual income [19, 39].

The finding of our study that young adults with disability have fewer episodes of alcohol intoxication than their able-bodied peers are in contrast to research reported elsewhere. A review of the literature showed that adolescents and young adults with special health-care needs are at greater risk of engaging in risk behaviour such as smoking and use of alcohol and drugs [41].

This study indicated that adolescent females with disability had a lower loneliness score and higher scholastic competence than adolescent boys. Gender differences in loneliness might be explained by different patterns of social interaction in boys and girls. Boys, to a larger extent than girls, socialize through sports and physical activity, which might be limited by their disability; however, this needs further investigation [42]. Higher scholastic achievement in adolescent girls is found in general [43]. However, the association between scholastic achievement and perceived scholastic competence is not entirely clear, and other studies have found no significant gender differences in perceived scholastic competence in adolescence [44].

Our findings revealed that young adults with disability continue to have more mental health problems than young adults without disability, but no such associations were found for young adults with chronic conditions compared with those without persistent health challenges. However, the adolescents with chronic health conditions of asthma alone, asthma and allergy, or diabetes had significantly more depressive symptoms, more anxiety symptoms and were less satisfied with their appearance than those without chronic health conditions. These findings are mostly consistent with the literature [10, 11, 15, 45, 46]. One Norwegian study found that type 1 diabetes was not associated with more symptoms of mental health problems; however, these findings are in contrast to higher levels of mental health problems found in previous research [47].

Interestingly, the mental health problems of adolescents with a chronic health condition seem to diminish in young adulthood. Several studies on adolescents with elevated levels of depressive symptoms have concluded that they continue to show similar levels of depressive symptoms later in life [48]. However, Dekker and colleagues found different trajectories in a large prospective study; *high stable trajectories, increasing trajectories and also decreasing trajectories* [48]. One possible explanation for decreasing trajectories of depressive symptoms might be related to distance in time from the original risk factors (i.e., events such as being diagnosed with a chronic condition) [48]. It is reasonable to assume that as an adolescent with a chronic condition matures and adapts to the illness and learns to cope with the challenges, it becomes easier to accept the situation, and thus the mental health problems decrease. Another possible explanation is that the major chronic condition in our study was asthma or allergy. Knowing that many children grow out of their asthma or allergy, or that the symptoms become more manageable, might indicate that the severity of the illness decreases, followed by the effect on mental health.

Adolescents with a chronic health condition had significantly more mental health problems than their peers without a chronic condition. In our study, adolescents with a chronic health condition scored worse than their peers on three out of nine variables that measure mental health problems, while adolescents with disabilities scored significantly worse on eight out of the nine variables. This gives us a clear indication that adolescents with disabilities are at much higher risk of having mental health problems than adolescents with a chronic condition.

The main strength of this study is the longitudinal design, which allowed us to examine associations between mental health problems and persistent health challenges in the transition from adolescence to young adulthood. However, one limitation might be that we only followed about 25 % of the representative sample at T1. Even though most of the attrition was planned, and the attrition analyses showed some significant differences between those who dropped out and those who completed the study, the large proportion of drop-outs at follow-up could be a source of bias [49, 50].

The findings of this study must be interpreted with reference to the characteristics of our sample. ‘Persistent health challenges’ is a collective term representing chronic health conditions and disability. The operational definition of chronic conditions in this study was adolescents and young adults with diabetes, asthma or allergy, and for disability, a variety of conditions were reported by the informants; difficulties with speaking, reading and writing and having a physical disability such as impaired vision, hearing or movement disability. It is noteworthy that this study was based on self-reported data and required participants to have sufficient reading and writing skills. Therefore, the level of cognitive functioning is presumably high. That said, it is a limitation of the study that the persistent health challenges are self-reported and that the self-reports are not validated against accepted diagnostic criteria. Another limitation is that in our dataset, we have no information on the severity of the different conditions included, only on the presence of a condition. It is reasonable to assume that there is a wide variety in the severity of conditions in our sample, from mild to severe. In a population-based birth cohort study in Western Australia, the relationship between asthma and mental health problems was studied [51]. It was found that severe asthma was associated with significantly increased odds of affective, anxiety, somatic, oppositional defiant and conduct problems in adolescents. Mild asthma was not associated with heightened vulnerability to mental health problems. Thus, not knowing the severity of the conditions included as persistent health challenges in our study means that the findings should be interpreted with caution. However, given the significant association between persistent health challenges and mental health problems in this study, we can presume that the severity of the conditions reported has the power to impact the lives of adolescents and young adults.

## Conclusions

In this longitudinal study, we have shown that adolescents with a persistent health challenge have a greater risk of heightened levels of mental health problems. On average, adolescents with disability had more mental health problems than those with a chronic health condition. In addition, the problems continued into adulthood for adolescents with disability. Disability seems to be a much higher risk factor for developing and continuing to experience mental health problems than having a chronic health condition. As such, the findings of the current study are alarming, and should be followed up in longitudinal studies with various groups of disability. Further, it is important to develop and test interventions aimed at young people with disabilities to reduce mental health problems and increase social participation in the transition from adolescence to adulthood.

## Abbreviations

BASS: Body areas satisfaction scale
SES: Socio-economic status
